# 3,3′-Di-tert-butyl-2′-hydr­oxy-5,5′,6,6′-tetra­methyl­biphenyl-2-yl benzene­sulfonate

**DOI:** 10.1107/S1600536809020959

**Published:** 2009-06-10

**Authors:** Po-Sheng Chen, Chia-Her Lin, Bao-Tsan Ko

**Affiliations:** a Department of Chemistry, Chung Yuan Christian University, Chung-Li 320, Taiwan

## Abstract

In the title compound, C_30_H_38_O_4_S, the hydroxyl group bonded to one phenyl ring and an O atom of the benzene­sulfonate group attached to the other phenyl ring of the biphenyl backbone of the structure are involved in an intra­molecular O—H⋯O hydrogen bond. The dihedral angle between the planes of the two aromatic rings of the biphenyl unit is 70.4 (2)°.

## Related literature

For the use of the binolate ligand 5,5′,6,6′-tetra­methyl-3,3′-di-*tert*-butyl-1,1′-bi-2,2′-phenolate in ring-closing metathesis reactions, see: La *et al.* (1998 ▶); For binolate–metal complexes, see: Chisholm *et al.* (2003 ▶); Wu *et al.* (2008 ▶). For related structures: see: Solinas *et al.* (2007 ▶).
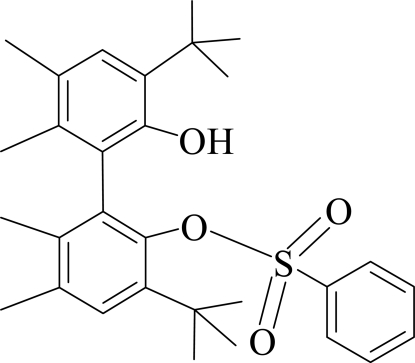


## Experimental

### 

#### Crystal data

C_30_H_38_O_4_S
*M* *_r_* = 494.66
Monoclinic, 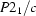

*a* = 9.9909 (7) Å
*b* = 13.3610 (11) Å
*c* = 20.2884 (16) Å
β = 93.428 (3)°
*V* = 2703.4 (4) Å^3^
*Z* = 4
Mo *K*α radiation
μ = 0.15 mm^−1^
*T* = 296 K
0.30 × 0.20 × 0.18 mm

#### Data collection

Bruker APEXII CCD diffractometer
Absorption correction: multi-scan (*SADABS*; Bruker, 2008 ▶) *T*_min_ = 0.964, *T*_max_ = 0.973
24566 measured reflections
6653 independent reflections
3695 reflections with *I* > 2σ(*I*)
*R*_int_ = 0.055

#### Refinement

*R*[*F* ^2^ > 2σ(*F* ^2^)] = 0.056
*wR*(*F* ^2^) = 0.164
*S* = 1.02
6653 reflections
316 parameters
H-atom parameters constrained
Δρ_max_ = 0.33 e Å^−3^
Δρ_min_ = −0.31 e Å^−3^

### 

Data collection: *APEX2* (Bruker, 2008 ▶); cell refinement: *SAINT-Plus* (Bruker, 2008 ▶); data reduction: *SAINT-Plus*; program(s) used to solve structure: *SHELXS97* (Sheldrick, 2008 ▶); program(s) used to refine structure: *SHELXL97* (Sheldrick, 2008 ▶); molecular graphics: *SHELXTL* (Sheldrick, 2008 ▶); software used to prepare material for publication: *SHELXTL*.

## Supplementary Material

Crystal structure: contains datablocks I, global. DOI: 10.1107/S1600536809020959/pv2156sup1.cif

Structure factors: contains datablocks I. DOI: 10.1107/S1600536809020959/pv2156Isup2.hkl

Additional supplementary materials:  crystallographic information; 3D view; checkCIF report

## Figures and Tables

**Table 1:** Hydrogen-bond geometry (Å, °)

*D*—H⋯*A*	*D*—H	H⋯*A*	*D*⋯*A*	*D*—H⋯*A*
O1—H1*A*⋯O4	0.82	2.37	3.107 (2)	150

**Fig. 1.**
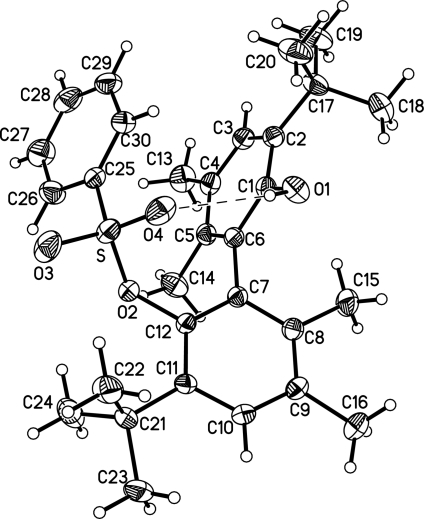
A view of the molecular structure of (I) with the atomn umbering scheme. Displacement ellipsoids are drawn at the 30% probability level.

**Fig. 2.**
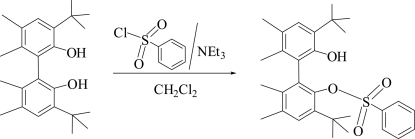
The formation of the title compound.

## supplementary crystallographic information

### Comment

During the last decade, bulky bis(phenolate) compounds have been attracting considerable attention, mainly due to their importance in the development of coordination chemistry related to catalytic applications. These bulky ligands are designed to provide a steric barrier around active metal center for minimizing the side reaction. Bulky binolate ligand, 5,5',6,6'-tetramethyl-3,3'-di-tert-butyl-1,1'-bi-2,2'-phenolate (BIPHEN^2-^) has proved to be important in ring-closing metathesis reactions in both its racemic and resolved forms (La *et al.*, 1998). Recently, a series of binolate metal complexes where the metal atoms are Li, Zn and Al have been synthesized, structurally characterized and studied for the catalytic activity of lactide polymerization (Chisholm *et al.*, 2003). Most recently, Wu and his co-workers, (Wu *et al.*, 2008) have also reported the magnesium complexes supported by mono-benzenesulfonylate phenol ligand and these complexes have been demonstrated as efficient initiators to catalyze ring-opening polymerization of lactides. Our group is interested in the synthesis and preparation of monovalent phenol derived from BIPHEN-H_2_. Here, we report the synthesis and crystal structure of the title compound, (I), a potential ligand for the preparation of magnesium and zinc complexes.

The structure of (I) is composed of a biphenyl moiety containing a benzenesulfonate and a hydroxyl group (Fig. 1). The dihedral angle between the planes of the two aromatic rings of the biphenyl unit is 70.4 (2)°. There is an intramolecular O—H···O hydrogen bond between the phenol and benzenesulfonate groups (Table 1). The distance of O4···H is substantially shorter than the van der Waals distance of 2.77 Å for the O and H distance. However, the distances O4···H and O4···O1 are around 0.3-0.4 Å longer than that found in the other mono-sulfonylated biaryl derivative with an intermolecular hydrogen-bonded motif (1.97 Å & 2.795 (2) Å; Solinas *et al.*, 2007).

### Experimental

The title compound (I) was synthesized by the following procedures (Scheme 2): 3,3'-di-tert-butyl-5,5',6,6'-tetramethylbiphenyl-2,2'-diol (1.24 g, 3.50 mmol), benzenesulfonyl chloride (0.45 mL, 3.50 mmol) and 4-(dimethylamino)pyridine (DMAP, 0.069 g, 0.57 mmol, catalyst) were dissolved in 50 mL of freshly distilled CH_2_Cl_2_ and the resulting solution cooled to 273 K. Neat triethylamine (NEt_3_, 0.6 mL, 3.85 mmol) was added dropwise to the solution, which was then stirred at ambient temperature for 48 h. The solution was filtered, and the filtrate was washed with 50 mL of water three times. The dichloromethane layer was collected and dried over anhydrous MgSO_4_ and filtered through Celite again to remove MgSO_4_. The resulting filtrate was then dried under vacuum to obtain the white solids (yield: 78 %). The resulting solid was crystallized from a toluene solution to yield colourless crystals of (I).

### Refinement

The H atoms were placed in idealized positions and constrained to ride on their parent atoms, with C—H = 0.93 and 0.96 Å allowing *U*_iso_(H) = 1.2 and 1.5*U*_eq_(C) for aryl and methyl groups, respectively; for hydroxy group, O—H = 0.82 Å and *U*_iso_(H) = 1.2*U*_eq_(O).

### Crystal data

**Table:**

C_30_H_38_O_4_S	*F*(000) = 1064
*M**_r_* = 494.66	*D*_x_ = 1.215 Mg m^−^^3^
Monoclinic, *P*2_1_/*c*	Mo *K*α radiation, λ = 0.71073 Å
Hall symbol: -P 2ybc	Cell parameters from 3976 reflections
*a* = 9.9909 (7) Å	θ = 2.5–22.8°
*b* = 13.3610 (11) Å	µ = 0.15 mm^−^^1^
*c* = 20.2884 (16) Å	*T* = 296 K
β = 93.428 (3)°	Columnar, colourless
*V* = 2703.4 (4) Å^3^	0.30 × 0.20 × 0.18 mm
*Z* = 4	

### Data collection

**Table:**

Bruker APEXII CCD diffractometer	6653 independent reflections
Radiation source: fine-focus sealed tube	3695 reflections with *I* > 2σ(*I*)
graphite	*R*_int_ = 0.055
φ and ω scans	θ_max_ = 28.4°, θ_min_ = 1.8°
Absorption correction: multi-scan (*SADABS*; Bruker, 2008)	*h* = −13→8
*T*_min_ = 0.964, *T*_max_ = 0.973	*k* = −17→17
24566 measured reflections	*l* = −26→26

### Refinement

**Table:**

Refinement on *F*^2^	Primary atom site location: structure-invariant direct methods
Least-squares matrix: full	Secondary atom site location: difference Fourier map
*R*[*F*^2^ > 2σ(*F*^2^)] = 0.056	Hydrogen site location: inferred from neighbouring sites
*wR*(*F*^2^) = 0.164	H-atom parameters constrained
*S* = 1.02	*w* = 1/[σ^2^(*F*_o_^2^) + (0.0774*P*)^2^ + 0.1593*P*] where *P* = (*F*_o_^2^ + 2*F*_c_^2^)/3
6653 reflections	(Δ/σ)_max_ < 0.001
316 parameters	Δρ_max_ = 0.33 e Å^−^^3^
0 restraints	Δρ_min_ = −0.31 e Å^−^^3^

### Special details

**Table:**

Experimental. ^1^H NMR (CDCl_3_, ppm): δ 7.20-7.46 (6H, m, ArH), 6.68 (1H, s, ArH), 5.00 (1H, s, OH), 2.32 (1H, s, CH_3_), 1.77 (1H, s, CH_3_), 1.74 (1H, s, CH_3_), 1.59 (9H, s, C(CH_3_)_3_), 1.50 (1H, s, CH_3_), 1.45 (9H, s, C(CH_3_)_3_).
Geometry. All esds (except the esd in the dihedral angle between two l.s. planes) are estimated using the full covariance matrix. The cell esds are taken into account individually in the estimation of esds in distances, angles and torsion angles; correlations between esds in cell parameters are only used when they are defined by crystal symmetry. An approximate (isotropic) treatment of cell esds is used for estimating esds involving l.s. planes.
Refinement. Refinement of F^2^ against ALL reflections. The weighted R-factor wR and goodness of fit S are based on F^2^, conventional R-factors R are based on F, with F set to zero for negative F^2^. The threshold expression of F^2^ > 2sigma(F^2^) is used only for calculating R-factors(gt) etc. and is not relevant to the choice of reflections for refinement. R-factors based on F^2^ are statistically about twice as large as those based on F, and R- factors based on ALL data will be even larger.

### Fractional atomic coordinates and isotropic or equivalent isotropic displacement parameters (Å^2^)

**Table:**

	*x*	*y*	*z*	*U*_iso_*/*U*_eq_	
S	0.36621 (6)	0.38183 (5)	0.17811 (3)	0.04538 (19)	
O1	0.65811 (15)	0.19585 (13)	0.25711 (8)	0.0520 (5)	
H1A	0.5798	0.2118	0.2612	0.078*	
O2	0.39067 (14)	0.32215 (11)	0.11199 (7)	0.0418 (4)	
O3	0.23658 (16)	0.42523 (15)	0.16868 (11)	0.0713 (6)	
O4	0.39560 (19)	0.31853 (13)	0.23306 (8)	0.0614 (5)	
C1	0.7141 (2)	0.25910 (17)	0.21289 (11)	0.0372 (5)	
C2	0.8289 (2)	0.31260 (18)	0.23429 (11)	0.0403 (5)	
C3	0.8784 (2)	0.37703 (18)	0.18794 (12)	0.0438 (6)	
H3B	0.9535	0.4151	0.2008	0.053*	
C4	0.8245 (2)	0.38863 (17)	0.12427 (12)	0.0425 (6)	
C5	0.7154 (2)	0.32891 (18)	0.10304 (11)	0.0401 (5)	
C6	0.6595 (2)	0.26372 (17)	0.14839 (11)	0.0357 (5)	
C7	0.5539 (2)	0.18961 (16)	0.12617 (10)	0.0354 (5)	
C8	0.5890 (2)	0.08878 (18)	0.12009 (11)	0.0397 (5)	
C9	0.4930 (2)	0.01987 (17)	0.09643 (11)	0.0403 (5)	
C10	0.3647 (2)	0.05417 (17)	0.07881 (11)	0.0401 (5)	
H10A	0.3020	0.0076	0.0625	0.048*	
C11	0.3231 (2)	0.15299 (17)	0.08380 (10)	0.0360 (5)	
C12	0.4217 (2)	0.21839 (16)	0.10941 (10)	0.0343 (5)	
C13	0.8876 (3)	0.4624 (2)	0.07860 (14)	0.0648 (8)	
H13A	0.9607	0.4961	0.1021	0.097*	
H13B	0.8217	0.5107	0.0632	0.097*	
H13C	0.9203	0.4272	0.0416	0.097*	
C14	0.6595 (3)	0.3336 (2)	0.03255 (12)	0.0587 (7)	
H14A	0.7095	0.3814	0.0086	0.088*	
H14B	0.5671	0.3536	0.0316	0.088*	
H14C	0.6663	0.2689	0.0125	0.088*	
C15	0.7310 (2)	0.0537 (2)	0.13777 (14)	0.0599 (7)	
H15A	0.7846	0.1096	0.1533	0.090*	
H15B	0.7684	0.0256	0.0994	0.090*	
H15C	0.7299	0.0039	0.1718	0.090*	
C16	0.5247 (2)	−0.08952 (19)	0.08847 (13)	0.0549 (7)	
H16A	0.4457	−0.1242	0.0716	0.082*	
H16B	0.5541	−0.1172	0.1305	0.082*	
H16C	0.5943	−0.0969	0.0582	0.082*	
C17	0.8981 (2)	0.2976 (2)	0.30302 (12)	0.0506 (6)	
C18	0.9429 (3)	0.1879 (2)	0.31150 (15)	0.0734 (9)	
H18A	0.8662	0.1448	0.3057	0.110*	
H18B	0.9845	0.1786	0.3549	0.110*	
H18C	1.0059	0.1720	0.2791	0.110*	
C19	1.0244 (3)	0.3626 (3)	0.31303 (15)	0.0776 (10)	
H19A	1.0002	0.4319	0.3083	0.116*	
H19B	1.0866	0.3452	0.2806	0.116*	
H19C	1.0652	0.3514	0.3564	0.116*	
C20	0.8038 (3)	0.3252 (3)	0.35657 (13)	0.0757 (9)	
H20A	0.7767	0.3938	0.3513	0.114*	
H20B	0.8492	0.3164	0.3992	0.114*	
H20C	0.7261	0.2829	0.3529	0.114*	
C21	0.1775 (2)	0.18286 (18)	0.06284 (12)	0.0428 (6)	
C22	0.1001 (2)	0.2033 (2)	0.12392 (14)	0.0609 (8)	
H22A	0.1426	0.2567	0.1489	0.091*	
H22B	0.0993	0.1440	0.1506	0.091*	
H22C	0.0097	0.2220	0.1107	0.091*	
C23	0.1028 (2)	0.0976 (2)	0.02594 (15)	0.0625 (8)	
H23A	0.0127	0.1183	0.0137	0.094*	
H23B	0.1009	0.0399	0.0540	0.094*	
H23C	0.1480	0.0812	−0.0131	0.094*	
C24	0.1724 (3)	0.2737 (2)	0.01644 (15)	0.0714 (9)	
H24A	0.2181	0.3290	0.0379	0.107*	
H24B	0.0806	0.2917	0.0057	0.107*	
H24C	0.2152	0.2572	−0.0233	0.107*	
C25	0.4840 (2)	0.47824 (17)	0.17560 (11)	0.0402 (5)	
C26	0.4767 (3)	0.5430 (2)	0.12214 (13)	0.0592 (7)	
H26A	0.4130	0.5331	0.0874	0.071*	
C27	0.5643 (3)	0.6219 (2)	0.12097 (16)	0.0681 (8)	
H27A	0.5614	0.6648	0.0848	0.082*	
C28	0.6552 (3)	0.6378 (2)	0.17246 (16)	0.0652 (8)	
H28A	0.7140	0.6917	0.1715	0.078*	
C29	0.6605 (3)	0.5750 (2)	0.22544 (15)	0.0627 (8)	
H29A	0.7223	0.5869	0.2607	0.075*	
C30	0.5747 (2)	0.4935 (2)	0.22742 (12)	0.0492 (6)	
H30A	0.5791	0.4502	0.2634	0.059*	

### Atomic displacement parameters (Å^2^)

**Table:**

	*U*^11^	*U*^22^	*U*^33^	*U*^12^	*U*^13^	*U*^23^
S	0.0427 (3)	0.0365 (4)	0.0576 (4)	−0.0017 (3)	0.0083 (3)	−0.0061 (3)
O1	0.0436 (9)	0.0635 (12)	0.0487 (10)	−0.0086 (8)	0.0011 (7)	0.0110 (9)
O2	0.0448 (8)	0.0324 (9)	0.0471 (9)	0.0014 (7)	−0.0058 (7)	−0.0024 (7)
O3	0.0406 (10)	0.0567 (12)	0.1176 (17)	0.0051 (9)	0.0124 (10)	−0.0200 (12)
O4	0.0897 (14)	0.0449 (11)	0.0511 (11)	−0.0069 (10)	0.0174 (9)	0.0024 (9)
C1	0.0339 (11)	0.0369 (13)	0.0409 (13)	0.0006 (9)	0.0034 (9)	0.0030 (10)
C2	0.0316 (11)	0.0422 (14)	0.0468 (14)	0.0012 (10)	0.0004 (10)	−0.0057 (11)
C3	0.0319 (11)	0.0409 (14)	0.0584 (16)	−0.0040 (10)	0.0019 (10)	−0.0043 (12)
C4	0.0364 (11)	0.0364 (13)	0.0553 (15)	−0.0015 (10)	0.0076 (10)	0.0019 (11)
C5	0.0363 (11)	0.0396 (14)	0.0443 (13)	0.0030 (10)	0.0020 (10)	0.0005 (11)
C6	0.0306 (10)	0.0349 (13)	0.0414 (13)	0.0003 (9)	0.0008 (9)	−0.0021 (10)
C7	0.0331 (10)	0.0340 (12)	0.0389 (12)	0.0004 (9)	0.0013 (9)	−0.0002 (10)
C8	0.0376 (11)	0.0406 (14)	0.0409 (13)	0.0027 (10)	0.0034 (9)	−0.0022 (11)
C9	0.0467 (13)	0.0344 (13)	0.0405 (13)	0.0005 (10)	0.0086 (10)	−0.0035 (10)
C10	0.0416 (12)	0.0361 (13)	0.0424 (13)	−0.0059 (10)	0.0012 (10)	−0.0046 (11)
C11	0.0378 (11)	0.0376 (13)	0.0322 (11)	−0.0017 (10)	−0.0008 (9)	−0.0033 (10)
C12	0.0380 (11)	0.0292 (12)	0.0354 (12)	0.0001 (9)	0.0011 (9)	−0.0002 (9)
C13	0.0617 (17)	0.0595 (19)	0.0741 (19)	−0.0114 (14)	0.0112 (14)	0.0113 (15)
C14	0.0577 (15)	0.074 (2)	0.0443 (15)	−0.0078 (14)	0.0011 (12)	0.0079 (14)
C15	0.0458 (14)	0.0536 (17)	0.0795 (19)	0.0144 (12)	−0.0017 (13)	−0.0046 (15)
C16	0.0606 (16)	0.0386 (15)	0.0667 (17)	0.0049 (12)	0.0130 (13)	−0.0053 (13)
C17	0.0393 (12)	0.0666 (18)	0.0446 (14)	−0.0009 (12)	−0.0078 (10)	−0.0047 (13)
C18	0.0639 (17)	0.081 (2)	0.073 (2)	0.0148 (16)	−0.0195 (15)	0.0068 (17)
C19	0.0595 (17)	0.100 (3)	0.070 (2)	−0.0212 (17)	−0.0197 (15)	−0.0072 (18)
C20	0.0661 (18)	0.114 (3)	0.0466 (16)	0.0055 (18)	−0.0005 (14)	−0.0178 (17)
C21	0.0334 (11)	0.0445 (14)	0.0493 (14)	−0.0013 (10)	−0.0077 (10)	−0.0032 (11)
C22	0.0354 (12)	0.074 (2)	0.0736 (19)	−0.0008 (13)	0.0059 (12)	−0.0162 (16)
C23	0.0447 (14)	0.0646 (19)	0.0760 (19)	−0.0079 (13)	−0.0155 (13)	−0.0168 (15)
C24	0.0560 (16)	0.073 (2)	0.082 (2)	−0.0045 (15)	−0.0219 (15)	0.0193 (17)
C25	0.0418 (12)	0.0325 (13)	0.0460 (13)	0.0005 (10)	−0.0003 (10)	−0.0050 (11)
C26	0.0673 (17)	0.0497 (17)	0.0584 (17)	−0.0112 (14)	−0.0145 (13)	0.0060 (14)
C27	0.081 (2)	0.0491 (18)	0.074 (2)	−0.0146 (15)	0.0029 (16)	0.0102 (15)
C28	0.0588 (17)	0.0476 (18)	0.090 (2)	−0.0145 (13)	0.0143 (16)	−0.0160 (17)
C29	0.0523 (15)	0.0607 (19)	0.073 (2)	−0.0030 (14)	−0.0115 (13)	−0.0236 (17)
C30	0.0505 (14)	0.0503 (16)	0.0462 (14)	0.0018 (12)	−0.0036 (11)	−0.0071 (12)

### Geometric parameters (Å, °)

**Table:**

S—O4	1.4161 (18)	C16—H16B	0.9600
S—O3	1.4215 (18)	C16—H16C	0.9600
S—O2	1.5920 (16)	C17—C20	1.526 (4)
S—C25	1.748 (2)	C17—C19	1.535 (4)
O1—C1	1.376 (3)	C17—C18	1.538 (4)
O1—H1A	0.8200	C18—H18A	0.9600
O2—C12	1.422 (3)	C18—H18B	0.9600
C1—C6	1.389 (3)	C18—H18C	0.9600
C1—C2	1.398 (3)	C19—H19A	0.9600
C2—C3	1.388 (3)	C19—H19B	0.9600
C2—C17	1.532 (3)	C19—H19C	0.9600
C3—C4	1.378 (3)	C20—H20A	0.9600
C3—H3B	0.9300	C20—H20B	0.9600
C4—C5	1.397 (3)	C20—H20C	0.9600
C4—C13	1.516 (3)	C21—C22	1.524 (3)
C5—C6	1.407 (3)	C21—C23	1.532 (3)
C5—C14	1.505 (3)	C21—C24	1.535 (4)
C6—C7	1.497 (3)	C22—H22A	0.9600
C7—C12	1.398 (3)	C22—H22B	0.9600
C7—C8	1.399 (3)	C22—H22C	0.9600
C8—C9	1.394 (3)	C23—H23A	0.9600
C8—C15	1.517 (3)	C23—H23B	0.9600
C9—C10	1.388 (3)	C23—H23C	0.9600
C9—C16	1.506 (3)	C24—H24A	0.9600
C10—C11	1.390 (3)	C24—H24B	0.9600
C10—H10A	0.9300	C24—H24C	0.9600
C11—C12	1.394 (3)	C25—C30	1.362 (3)
C11—C21	1.544 (3)	C25—C26	1.386 (3)
C13—H13A	0.9600	C26—C27	1.371 (4)
C13—H13B	0.9600	C26—H26A	0.9300
C13—H13C	0.9600	C27—C28	1.360 (4)
C14—H14A	0.9600	C27—H27A	0.9300
C14—H14B	0.9600	C28—C29	1.362 (4)
C14—H14C	0.9600	C28—H28A	0.9300
C15—H15A	0.9600	C29—C30	1.389 (4)
C15—H15B	0.9600	C29—H29A	0.9300
C15—H15C	0.9600	C30—H30A	0.9300
C16—H16A	0.9600		
			
O4—S—O3	119.60 (12)	H16B—C16—H16C	109.5
O4—S—O2	109.22 (10)	C20—C17—C2	110.6 (2)
O3—S—O2	105.99 (10)	C20—C17—C19	107.8 (2)
O4—S—C25	110.79 (11)	C2—C17—C19	111.7 (2)
O3—S—C25	107.77 (11)	C20—C17—C18	109.8 (2)
O2—S—C25	101.95 (10)	C2—C17—C18	109.8 (2)
C1—O1—H1A	109.5	C19—C17—C18	107.1 (2)
C12—O2—S	124.34 (13)	C17—C18—H18A	109.5
O1—C1—C6	119.32 (19)	C17—C18—H18B	109.5
O1—C1—C2	118.01 (19)	H18A—C18—H18B	109.5
C6—C1—C2	122.6 (2)	C17—C18—H18C	109.5
C3—C2—C1	115.2 (2)	H18A—C18—H18C	109.5
C3—C2—C17	122.5 (2)	H18B—C18—H18C	109.5
C1—C2—C17	122.2 (2)	C17—C19—H19A	109.5
C4—C3—C2	124.7 (2)	C17—C19—H19B	109.5
C4—C3—H3B	117.7	H19A—C19—H19B	109.5
C2—C3—H3B	117.7	C17—C19—H19C	109.5
C3—C4—C5	118.6 (2)	H19A—C19—H19C	109.5
C3—C4—C13	119.5 (2)	H19B—C19—H19C	109.5
C5—C4—C13	121.9 (2)	C17—C20—H20A	109.5
C4—C5—C6	119.0 (2)	C17—C20—H20B	109.5
C4—C5—C14	120.5 (2)	H20A—C20—H20B	109.5
C6—C5—C14	120.5 (2)	C17—C20—H20C	109.5
C1—C6—C5	119.66 (19)	H20A—C20—H20C	109.5
C1—C6—C7	118.98 (19)	H20B—C20—H20C	109.5
C5—C6—C7	120.87 (19)	C22—C21—C23	105.9 (2)
C12—C7—C8	118.76 (19)	C22—C21—C24	110.9 (2)
C12—C7—C6	122.01 (19)	C23—C21—C24	106.9 (2)
C8—C7—C6	119.20 (18)	C22—C21—C11	109.74 (18)
C9—C8—C7	119.73 (19)	C23—C21—C11	111.5 (2)
C9—C8—C15	119.5 (2)	C24—C21—C11	111.68 (19)
C7—C8—C15	120.8 (2)	C21—C22—H22A	109.5
C10—C9—C8	118.4 (2)	C21—C22—H22B	109.5
C10—C9—C16	119.3 (2)	H22A—C22—H22B	109.5
C8—C9—C16	122.3 (2)	C21—C22—H22C	109.5
C9—C10—C11	124.8 (2)	H22A—C22—H22C	109.5
C9—C10—H10A	117.6	H22B—C22—H22C	109.5
C11—C10—H10A	117.6	C21—C23—H23A	109.5
C10—C11—C12	114.52 (19)	C21—C23—H23B	109.5
C10—C11—C21	120.42 (19)	H23A—C23—H23B	109.5
C12—C11—C21	125.1 (2)	C21—C23—H23C	109.5
C11—C12—C7	123.7 (2)	H23A—C23—H23C	109.5
C11—C12—O2	118.27 (18)	H23B—C23—H23C	109.5
C7—C12—O2	117.67 (18)	C21—C24—H24A	109.5
C4—C13—H13A	109.5	C21—C24—H24B	109.5
C4—C13—H13B	109.5	H24A—C24—H24B	109.5
H13A—C13—H13B	109.5	C21—C24—H24C	109.5
C4—C13—H13C	109.5	H24A—C24—H24C	109.5
H13A—C13—H13C	109.5	H24B—C24—H24C	109.5
H13B—C13—H13C	109.5	C30—C25—C26	120.8 (2)
C5—C14—H14A	109.5	C30—C25—S	120.40 (19)
C5—C14—H14B	109.5	C26—C25—S	118.62 (18)
H14A—C14—H14B	109.5	C27—C26—C25	119.3 (2)
C5—C14—H14C	109.5	C27—C26—H26A	120.3
H14A—C14—H14C	109.5	C25—C26—H26A	120.3
H14B—C14—H14C	109.5	C28—C27—C26	120.3 (3)
C8—C15—H15A	109.5	C28—C27—H27A	119.9
C8—C15—H15B	109.5	C26—C27—H27A	119.9
H15A—C15—H15B	109.5	C27—C28—C29	120.2 (3)
C8—C15—H15C	109.5	C27—C28—H28A	119.9
H15A—C15—H15C	109.5	C29—C28—H28A	119.9
H15B—C15—H15C	109.5	C28—C29—C30	120.7 (3)
C9—C16—H16A	109.5	C28—C29—H29A	119.6
C9—C16—H16B	109.5	C30—C29—H29A	119.6
H16A—C16—H16B	109.5	C25—C30—C29	118.6 (3)
C9—C16—H16C	109.5	C25—C30—H30A	120.7
H16A—C16—H16C	109.5	C29—C30—H30A	120.7

### Hydrogen-bond geometry (Å, °)

**Table:**

*D*—H···*A*	*D*—H	H···*A*	*D*···*A*	*D*—H···*A*
O1—H1A···O4	0.82	2.37	3.107 (2)	150
